# Influence of secreted frizzled receptor protein 1 (SFRP1) on neoadjuvant chemotherapy in triple negative breast cancer does not rely on WNT signaling

**DOI:** 10.1186/1476-4598-13-174

**Published:** 2014-07-17

**Authors:** Christof Bernemann, Carolin Hülsewig, Christian Ruckert, Sarah Schäfer, Lena Blümel, Georg Hempel, Martin Götte, Burkhard Greve, Peter J Barth, Ludwig Kiesel, Cornelia Liedtke

**Affiliations:** 1 Translational Tumor Biology Group, Albert-Schweitzer Campus 1 A1, 48149, Münster, Germany; 2 Department of Gynecology and Obstetrics, University Hospital Münster, Albert-Schweitzer Campus 1 A1, 48149, Münster, Germany; 3 University of Münster, Medical Faculty, Domagkstr, 48149, Münster, Germany; 4 Institute of Human Genetics, University of Münster, Vesaliusweg 12-14, 48149 Münster, Germany; 5 Internal Medicine, Marienhospital, Rochusstr., 40237, Düsseldorf, Germany; 6 Department of Pharmaceutical and Medical Chemistry, University of Münster, Corrensstrasse 48, 48149 Münster, Germany; 7 Department of Radiotherapy – Radiooncology, University Hospital Münster, Albert-Schweitzer Campus 1 A1, 48149 Münster, Germany; 8 Gerhard-Domagk-Institute of Pathology, University Hospital Münster, Albert-Schweitzer Campus 1 A1, 48149 Münster, Germany; 9 Department of Gynecology and Obstetrics, University Hospital Schleswig Holstein, Campus Lübeck, Ratzeburger Allee 160, 23538 Lübeck, Germany

**Keywords:** Triple negative breast cancer, SFRP1, Chemotherapy sensitivity, Prognostic marker

## Abstract

**Background:**

Triple negative breast cancer (TNBC) is characterized by lack of expression of both estrogen and progesterone receptor as well as lack of overexpression or amplification of HER2. Despite an increased probability of response to chemotherapy, many patients resistant to current chemotherapy regimens suffer from a worse prognosis compared to other breast cancer subtypes. However, molecular determinants of response to chemotherapy specific to TNBC remain largely unknown. Thus, there is a high demand for biomarkers potentially stratifying triple negative breast cancer patients for neoadjuvant chemotherapies or alternative therapies.

**Methods:**

In order to identify genes correlating with both the triple negative breast cancer subtype as well as response to neoadjuvant chemotherapy we employed publicly available gene expression profiles of patients, which had received neoadjuvant chemotherapy. Analysis of tissue microarrays as well as breast cancer cell lines revealed correlation to the triple negative breast cancer subtype. Subsequently, effects of siRNA-mediated knockdown on response to standard chemotherapeutic agents as well as radiation therapy were analyzed. Additionally, we evaluated the molecular mechanisms by which SFRP1 alters the carcinogenic properties of breast cancer cells.

**Results:**

SFRP1 was identified as being significantly overexpressed in TNBC compared to other breast cancer subtypes. Additionally, SFRP1 expression is significantly correlated with an increased probability of positive response to neoadjuvant chemotherapy. Knockdown of SFRP1 in triple negative breast cancer cells renders the cells more resistant to standard chemotherapy. Moreover, tumorigenic properties of the cells are modified by knockdown, as shown by both migration or invasion capacity as well reduced apoptotic events. Surprisingly, we found that these effects do not rely on Wnt signaling. Furthermore, we show that pro-apoptotic as well as migratory pathways are differentially regulated after SFRP1 knockdown.

**Conclusion:**

We could firstly show that SFRP1 strongly correlates with the triple negative breast cancer subtype and secondly, that SFRP1 might be used as a marker stratifying patients to positively respond to neoadjuvant chemotherapy. The mechanisms by which tumor suppressor SFRP1 influences carcinogenic properties of cancer cells do not rely on Wnt signaling, thereby demonstrating the complexity of tumor associated signaling pathways.

## Background

Triple negative breast cancer (TNBC) is defined by the lack of both estrogen receptor (ER) and progesterone receptor (PR) expression as well as overexpression or amplification of the *human epidermal growth factor receptor* HER2 [1-3]. Patients suffering from TNBC are not eligible for endocrine or HER2 targeted therapies, thus rendering chemotherapy the only therapeutic option, which may be accompanied by antiangiogenic approaches such as bevacizumab in the palliative setting [2,4,5]. Up to 15% of all breast cancer patients are diagnosed with TNBC [3]. Due to high recurrence rates and an increased risk of visceral and cerebral metastases these patients have a poorer prognosis in comparison to other breast cancer subtypes [6-8]. However, patients suffering from TNBC do have an increased probability of positive response to anthracycline/taxane- containing neoadjuvant chemotherapy. Thus, by achieving a pathologic complete response after neodajuvant chemotherapy the prognosis is as good as in other breast cancer subtypes [9]. Consequently, as chemotherapy sensitivity is one of the most important prognostic factors, it is inevitable to identify biomarkers and potential mediators of chemotherapy sensitivity in patients with TNBC.

The scientific goal of this study was to identify biomarkers, which may serve as mediators of chemotherapy sensitivity in TNBC. By using global gene expression profiles of patients receiving neoadjuvant chemotherapy we could identify *secreted frizzled receptor protein 1* (SFRP1) as being correlated with the triple negative breast cancer subtype. Furthermore, we found a positive correlation of SFRP1 expression and response to neoadjuvant chemotherapy.

SFRP1 has been described to antagonize canonical Wnt signaling by binding to Wnt proteins or Wnt receptors, thereby inhibiting their downstream signaling activity [10]. In a plethora of solid tumors, including colorectal cancer, ovarian cancer, prostate cancer and lung cancer, it has been shown that SFRP1 is inactivated by promoter hypermethylation [11-15]. In breast cancer, hypermethylation of the SFRP1 promoter has been correlated to poor prognosis, presumably due to elevated levels of Wnt signaling [16,17].

By analyzing the molecular role of SFRP1 in triple negative breast cancer cells via siRNA mediated knockdown we found changes in carcinogenic properties of breast cancer cells, e.g. increased migration and invasion potential as well as reduced apoptotic events. Furthermore, we observed an increased resistance to standard cytostatic agents. Surprisingly, although SFRP1 is known to act via canonical Wnt signaling, our data suggests that its influence on triple negative breast cancer cells is apparently not mediated via this pathway.

In summary, we could show that tumor suppressor and Wnt signaling antagonist SFRP1 is correlated with the most aggressive subtype of breast cancer, i.e. triple negative breast cancer; but also with positive response to neoadjuvant chemotherapy. This makes SFRP1 a potential biomarker for future stratification of triple negative breast cancer patients. Additionally, SFRP1 seems to be involved in regulatory processes necessary for tumorigenic cancer cells, e.g. regulation of apoptosis as well as migration and adhesion processes. Surprisingly, however, these mechanisms are not mediated by canonical Wnt signaling.

## Results

### SFRP1 expression correlates with the TNBC subtype and response to neoadjuvant chemotherapy

**Figure 1:**
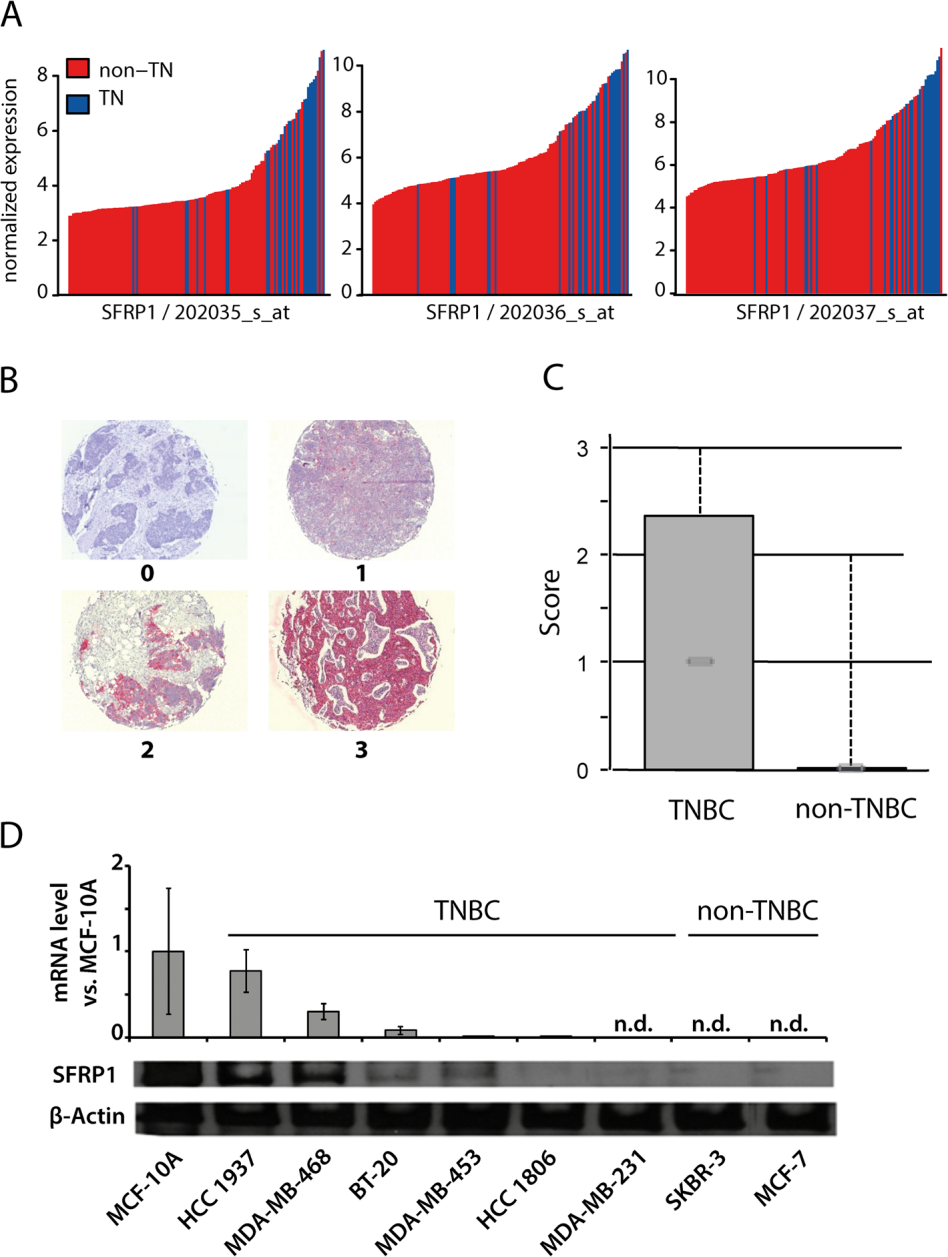
**Expression of SFRP1 correlates with the triple negative breast cancer subtype. A)** SFRP1 was found to be upregulated in TNBC compared to non-TNBC; shown here are all three probesets 202035_s_at, 202036_s_at and 202037_s_at (TN = triple negative). **B)** Expression of SFRP1 in breast cancer tissue specimens of 362 patients (TNBC = 37patients); scores 0 (negative), 1 (weak positive), 2 (positive), 3 (strong positive). **C)** Boxplot analysis of immunohistochemical staining. **D)** Protein and mRNA levels of SFRP1 in different breast (cancer) cell lines (non-tumorigenic epithelial cell line: MCF-10A, TNBC cell lines: HCC1937, MDA-MB-468, BT-20, MDA-MB-453, HCC 1806 and MDA-MB-231; non-TNBC cell lines SKBR-3 and MCF-7) (n.d. = not detected).

In order to identify genes involved in chemotherapy response in patients suffering from TNBC we made use of a published dataset analyzing global gene expression profiles of breast cancer patients receiving neoadjuvant chemotherapy [18]. This dataset combines pretreatment gene expression profiles with response to neoadjuvant chemotherapy, e.g. showing either residual disease (n = 99) or pathologic complete response (n = 34). By analyzing both gene expression profiles as well as response to neoadjuvant chemotherapy we could firstly, show that SFRP1 expression correlates with the triple negative breast cancer subtype and secondly, demonstrate association between expression of SFRP1 and positive response to neoadjuvant chemotherapy, i.e. achievement of a pathologic complete response (Figure 1A, Tables 1, 2).We next sought to analyze the expression of SFRP1 in breast cancer tissue specimen. Therefore, we performed immunohistochemical analyses of tissue microarrays of breast cancer patients previously categorized as being either TNBC or non-TNBC. We detected different levels of SFRP1 expression mostly located in the tumor tissue (Figure 1B). When analyzing the distinct scores of expression of SFRP1 we found strong correlation of SFRP1 expression and the triple negative breast cancer subtype (Figure 1C).In addition, by using quantitative real time PCR and western blot analyses we found expression of SFRP1 in 5 out of 6 triple negative breast cancer cell lines (HCC-1937, MDA-MB-468, BT-20, MDA-MB-453 and HCC-1806), whereas no expression was detected in the TNBC cell line MDA-MB-231 as well as the non-TNBC cell lines SKBR-3 or MCF-7 (Figure 1D). These results clearly demonstrate a correlation of expression of SFRP1 and the triple negative breast cancer subtype. Additionally, there seems to be a link between the response to neoadjuvant chemotherapy and expression of SFRP1.

**Table 1:** Expression of SFRP1 correlates with the triple negative breast cancer subtype

**Probeset**	**Gene ID**	**Adjusted p-value**	**Upregulated**	**Mean non-TN**	**Mean TN**
202035_s_at	SFRP1	1.4 10E - 5	TNBC	3.844	5.879
202036_s_at	SFRP1	5.5 10E - 6	TNBC	5.734	7.966
202037_s_at	SFRP1	3.0 10E - 6	TNBC	6.187	8.424

**Table 2:** Expression of SFRP1 correlates with positive response to neoadjuvant chemotherapy

**Probeset**	**Gene ID**	**Adjusted p-value**	**Upregulated**	**Mean RD**	**Mean pCR**
202035_s_at	SFRP1	0.017	pCR	4.993	6.833
202036_s_at	SFRP1	0.035	pCR	7.116	8.881
202037_s_at	SFRP1	0.021	pCR	7.553	9.364

### Knockdown of SFRP1 increases resistance against both chemotherapeutic agents as well as radiotherapy in triple negative breast cancer cells

**Figure 2:**
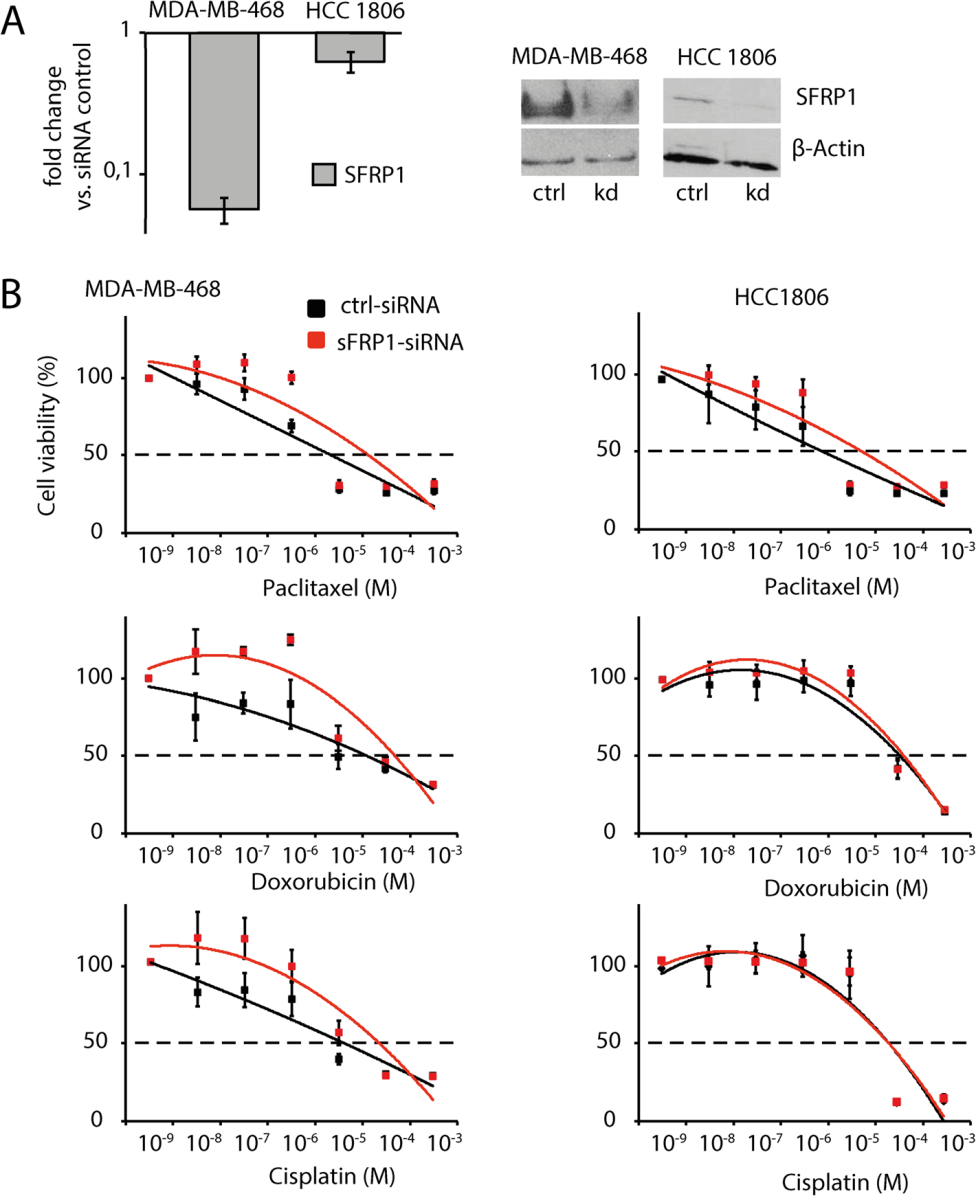
**Downregulation of SFRP1 renders triple negative breast cancer cells more resistant to standard chemotherapy. A)** mRNA (left) and protein (right) levels of SFRP1 after siRNA mediated knockdown, as determined by qPCR and Western-Blotting, respectively. **B)** Chemotherapy sensitivity of MDA-MB-468 and HCC-1806 after SFRP1 knockdown was determined using MTT cell viability assay of control vs. SFRP1 knockdown cells after treatment with cytotoxic agents (significance in MDA-MB-468 for paclitaxel ***p < 0.001 for 10 pM-1 nM, for doxorubicin ***p < 0.001 for 500 pM-50 nM and for cisplatin *p < 0.05 for 50 nM-50 μM; no significant changes were found in HCC-1806), error bars = SD, n = 3.

In order to analyze the role of SFRP1 in triple negative breast cancer cells, we performed siRNA-mediated knockdown of SFRP1 in the triple negative breast cancer cell lines MDA-MB-468 and HCC-1806. Efficiency of knockdown was proven via quantitative real-time PCR and Western Blot analysis (Figure 2A).We next sought to evaluate the influence of SFRP1 knockdown to standard triple negative breast cancer chemotherapy. Therefore, we analyzed the response to chemotherapeutic agents after SFRP1 knockdown by cell viability assay. Interestingly, downregulation of SFRP1 expression rendered triple negative breast cancer cell line MDA-MB-468 more resistant to the chemotherapeutic agents paclitaxel, cisplatinum and doxorubicin (Figure 2B, left column). Another triple negative breast cancer cell line, HCC-1806, showed only increased resistance to paclitaxel (Figure 2B, right column). Thus, the knockdown of SFRP1 renders cancer cells more resistant to standard chemotherapeutic treatment.

### Knockdown of SFRP1 enhances the carcinogenic properties of triple negative breast cancer cells

**Figure 3:**
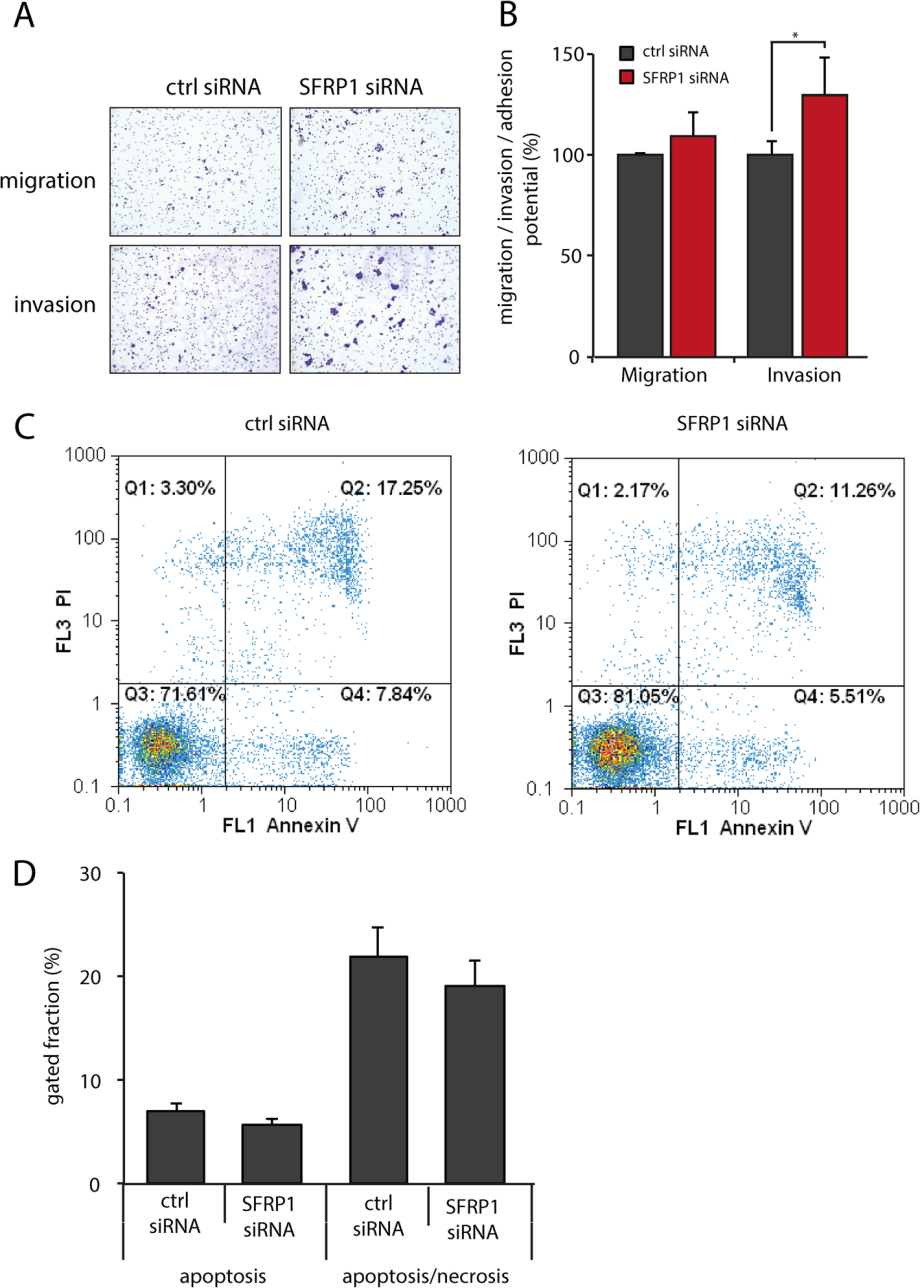
**Downregulation of SFRP1 changes carcinogenic properties of triple negative breast cancer cells. A)** Representative pictures of migration and invasion filters after SFRP1 knockdown in MDA-MB-468 cells. **B)** Quantitative analysis of migration and invasion assays after SFRP1 knockdown revealed increase in migration of about 15% and increase in invasion potential of about 30% ((*p < 0.05), error bars = SD, n = 3) **C/D)** Flow cytometry analysis using Annexin V staining showed a slight decrease of cells undergoing apoptotic (C: Q4) as well as late apoptotic or necrotic events (C: Q1 and Q2).

Next, we sought to explore the impact of SFRP1 on tumorigenic properties of breast cancer cells. Hence, we analyzed both migration as well as invasion potential of breast cancer cell line MDA-MB-468 after SFRP1 knockdown. The migratory potential was not significantly increased after knockdown. However, invasion through matrigel-coated membranes was significantly increased by about 30% after SFRP1 knockdown (Figure 3A, B (left, center)). Additionally, we thought whether the rate of apoptosis is influences upon SFRP1 knockdown. Therefore, we analyzed the amount of apoptotic and necrotic cells via flow cytometry. We found a slight decrease of apoptotic as well as necrotic cells after SFRP1 knockdown (Figure 3C, D). These results demonstrate a link between expression of SFRP1 and carcinogenic properties of breast cancer cells.

### The changes of tumorigenic potential of cells after SFRP1 knockdown do not rely on Wnt signaling

**Figure 4:**
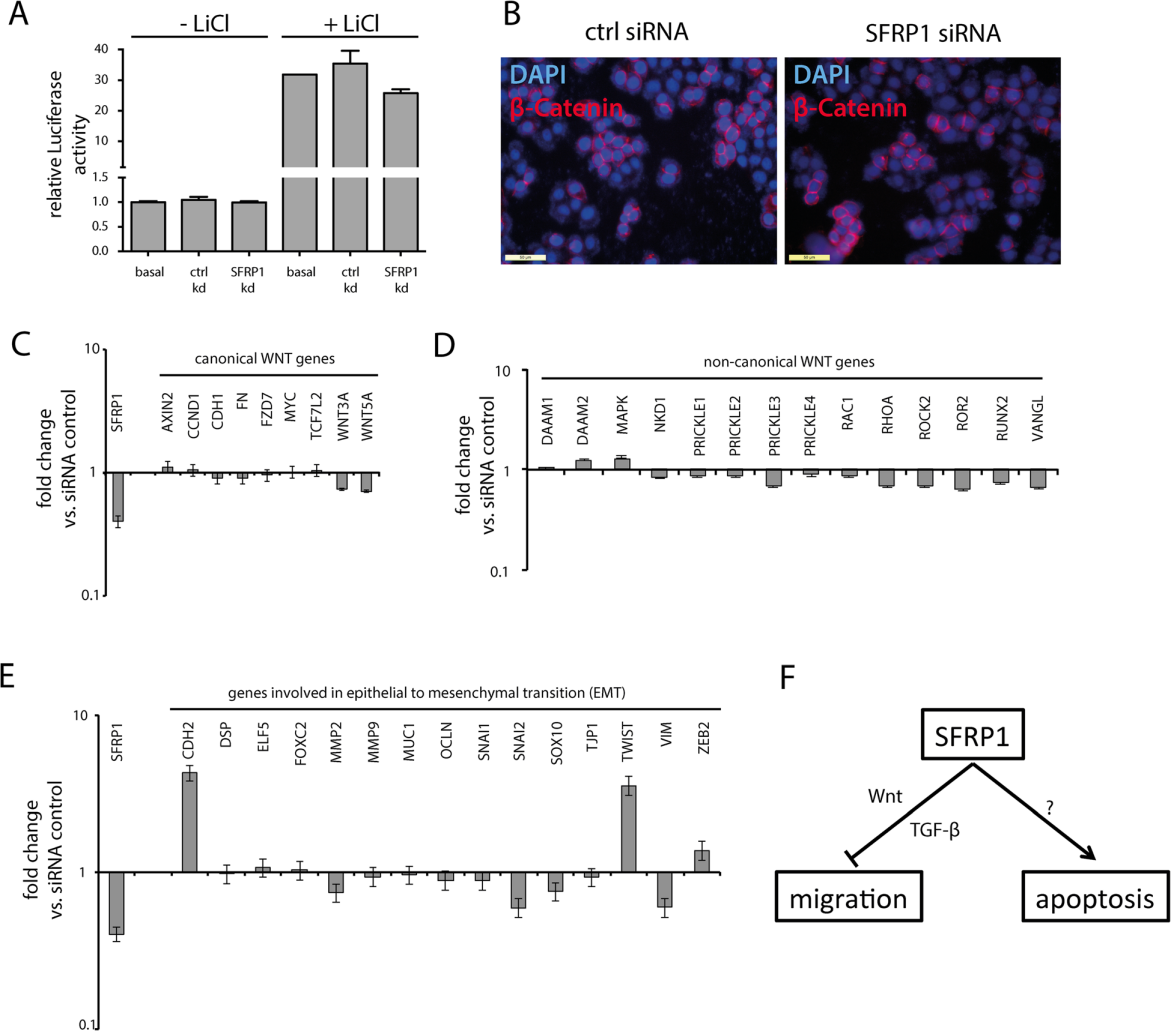
**Influence of SFRP1 on carcinogenic properties does not rely on canonical Wnt signaling. A)** Luciferase assays showed neither changes in Wnt signaling activity upon SFRP1 knockdown in breast cancer cells grown in standard medium (left 3 rows), nor in breast cancer cells grown in starvation medium followed by Wnt stimulation using LiCl treatment (right 3 rows). **B)** Immunofluorescence analysis showed no changes in cellular localization of β-Catenin after SFRP1 knockdown. C/D) mRNA levels of known canonical Wnt target genes **(C)** as well as members of the non-canonical Wnt signaling pathway **(D)** revealed no significant changes upon SFRP1 knockdown. **E)** mRNA levels of EMT related genes after SFRP1 knockdown. **F)** Model of action of SFRP1 in breast cancer.

SFRP1 is known to antagonize Wnt signaling activity via binding to either Wnt proteins or frizzled receptors, thereby blocking the intracellular signaling cascade [17]. Thus, we hypothesized that loss of SFRP1 might activate canonical Wnt signaling activity. Therefore, we performed a TOP-Flash/FOP-Flash luciferase assay in order to analyze the effects of SFRP1 knockdown on the triple negative breast cancer cell line MDA-MB-468. Surprisingly, we were not able to detect any changes in Wnt signaling activity of breast cancer cells after SFRP1 knockdown (Figure 4A (left)). As Wnt signaling activity is known to be very low in breast cancer cells, we made use of a known Wnt activator, LiCl, in order to stimulate Wnt signaling activity [19-21]. Treatment of cells with LiCl leads to a significant increase of Wnt signaling activity of about 30-fold. However, no significant changes were found in SFRP1 knockdown cells compared to control cells (Figure 4A (right)). Additionally, when analyzing the cellular localization of β-Catenin by immunofluorescence staining we were not able to detect any enhanced nuclear localization of β-Catenin after SFRP1 knockdown, typically a hallmark of activated Wnt signaling (Figure 4B).

We also performed global gene expression profile analysis via microarray analysis after SFRP1 knockdown in MDA-MB-468 cells. When analyzing effects on known canonical Wnt target genes, we were not able to detect any significant changes in gene expression (Table 3). In order to validate the microarray gene expression analysis data, we also performed quantitative real time PCR analysis. Although we found a decrease of approximately 60% in SFRP1 expression upon siRNA mediated knockdown, no significant changes of known Wnt target genes were observed (Figure 4C). Furthermore, we analyzed the expression levels of known members of the non-canonical Wnt signaling pathway [22-24]. However, we could not detect any significant changes in expression levels of these genes (Figure 4D). Since the Wnt signaling pathway is also correlated with the phenomenon of epithelial to mesenchymal transition, a process in which epithelial cells gain characteristics of mesenchymal cells, e.g. increased migratory or invasive potential, we also analyzed the expression levels of a plethora of known EMT related genes [25]. Although we found an increase in known EMT related genes N-Cadherin (CDH2) as well as TWIST, the majority of EMT genes remained unaffected after SFRP1 knockdown (Figure 4E). Thus, the effects of SFRP1 knockdown on carcinogenic properties on triple negative breast cancer cells do not appear to be mediated by elevated levels of canonical nor non-canonical Wnt signalling.

**Table 3:** Expression of known Wnt target genes in SFRP1-depleted MDA-MB-468 cells

**Name**	**SEQ_ID**	**Ratio**
TCF7L2	NM_030756	2.37
MMP7	NM_002423	2.28
FN	NM_054034	1.57
CDH1	AB025105	1.37
CDH1	AB025106	1.31
FZD7	BC015915	1.26
VEGF	AY263145	1.2
AXIN2	BC101533	1.19
VEGF	M27281	1.16
CLDN1	BC012471	1.16
VEGF	S85192	1.16
CDH1	NM_004360	1.16
AXIN2	NM_004655	1.13
CLDN1	NM_021101	1.08
JUN	BC006175	1.07
MYC	NM_002467	1.01
BGLAP	NM_199173	0.97
MYCBP	NM_012333	0.95
ID2	NM_002166	0.94
CCND1	NM_053056	0.94
CCND1	BC001501	0.94
PPARD	NM_001039694	0.94
MYCBP	BC008686	0.9
SFRP1	NM_003012	0.28

Only 2 of several known Wnt target genes show increased expression after SFRP1 knockdown (List of genes available from: The Wnt homepage; http://www.stanford.edu/group/nusselab/cgi-bin/wnt/target_genes).

### SFRP1 influences effects of cell adhesion and apoptosis

To gain insights into the mechanism underlying effects of SFRP1 knockdown on carcinogenic properties of triple negative breast cancer cells, we performed global gene expression profiling after SFRP1 knockdown followed by gene ontology analysis. When analyzing the list of genes showing upregulation of more than 30% after knockdown by using DAVID gene ontology database (http://david.abcc.ncifcrf.gov, [26]) we found several genes involved in cell adhesion, cell motion as well as signaling activity (Table 4). On the other hand, when analyzing genes showing downregulation of more than 25% after SFRP1 knockdown, we primarily found genes involved in positive regulation of apoptosis (Table 5), which is consistent with our observed decrease in apoptotic events in these cells (Figure 3C, D). Thus, SFRP1 knockdown positively regulates mechanisms of cell adhesion as well as the survival of cells presumably by inhibiting signals usually associated with apoptosis.

**Table 4:** Gene ontology analysis of genes showing upregulation of > 130% after SFRP1 knockdown in MDA-MB-468 cells

**Term**	**Count**	**p-Value**
Intracellular signaling cascade	68	7.40E-04
Positive regulation of transferase activity	20	1.20E-03
Cell motion	31	2.20E-03
Regulation of transferase activity	26	2.20E-03
Biological adhesion	41	2.80E-03
Cell adhesion	41	2.90E-03
Positive regulation of kinase activity	18	4.40E-03
Monosaccharide transport	6	4.70E-03
Regulation of kinase activity	24	5.40E-03
Cell migration	20	5.60E-03

**Table 5:** Gene ontology analysis of genes showing downregulation of < 75% after SFRP1 knockdown in MDA-MB-468 cells

**Term**	**Count**	**p-Value**
Negative regulation of cell proliferation	19	1.60E-05
Positive regulation of apoptosis	20	5.00E-05
Positive regulation of programmed cell death	20	5.50E-05
Positive regulation of cell death	20	5.90E-05
Regulation of cellular localization	14	1.50E-04
Regulation of apoptosis	28	1.60E-04
Regulation of programmed cell death	28	1.90E-04
Induction of apoptosis by intracellular signals	7	2.00E-04
Regulation of cell death	28	2.00E-04

## Discussion

Triple negative breast cancer is the most aggressive breast cancer subtype associated with poor prognosis as well as high recurrence rates. Since patients suffering from TNBC do have an unfavorable prognosis mostly due to limited therapeutic options, this cancer subtype has gained much attention regarding the development of novel targeted therapies. However, a high number of TNBC patients do positively respond to neoadjuvant chemotherapy. As a result, achieving a pathologic complete response (pCR) is correlated with good prognosis similar to other breast cancer subtypes [9]. Thus, the identification of patients responding to neoadjuvant chemotherapy would greatly improve the therapeutic options for TNBC. Other TNBC patients, however, would need to be treated differently, e.g. by anti-angiogenic treatment.

By using a published dataset analyzing differential gene expression profiles as well as response to neoadjuvant chemotherapeutic treatment of breast cancer patients we could show a correlation of SFRP1 expression and the triple negative breast cancer subtype.

We could demonstrate that breast cancer cell lines showing increased SFRP1 expression are associated with the triple negative phenotype, similar to published results showing higher expression in basal like cancer cell lines compared to luminal cell lines [14]. Interestingly, we mainly found increased expression of SFRP1 in triple negative breast cancer cell lines, which molecularly belong to basal A subtype described by Neve et al. (HCC1937, MDA-MB-468 and BT20) [27]. Cell lines of basal A subtype display more epithelial characteristics, whereas basal B cell lines are shown to be more invasive presumably due to spindle-like morphology displaying mesenchymal as well as stem/progenitor-like characteristics.

Furthermore, the basal A subtype has been correlated with increased response to neoadjuvant chemotherapy when compared to the basal B subtype [28,29]. In line with these observations we could show that expression of SFRP1 is also correlated with the achievement of a pathologic complete response after neoadjuvant chemotherapy. Thus, SFRP1 might become a useful biomarker to stratify triple negative breast cancer patients, which might benefit from neoadjuvant treatment. However, for clinical applications using SFRP1 expression as a prognostic biomarker, a proper platform is needed e.g. immunohistochemistry (IHC) or fluorescent in situ hybridization (FISH) followed by conversion into dichotomous status [30].

SFRP1 belongs to the family of 5 secreted frizzled receptor proteins, which show homology to the frizzled proteins, surface receptors for Wnt proteins [31]. SFRP1 has been described to antagonize canonical Wnt signaling by binding to either Wnt ligand proteins or frizzled receptors, thereby inhibiting the downstream signaling cascade [17]. SFRP1 has been linked to a number of solid tumors, e.g. colon cancer, ovarian cancer, prostate cancer or breast cancer [11-17]. It has been shown that the SFRP1 promoter is hypermethylated in these entities, thereby inactivating SFRP1 expression and its protein translation.

When analyzing the influence of loss of SFRP1 in triple negative breast cancer cells we found an increase of tumor-associated characteristics, e.g. increase in migration and invasion capacity, reduced apoptotic events as well as resistance to cytotoxic chemotherapy. Increased Wnt signaling is known to regulate tumor progression mechanisms as well as resistance to chemotherapy or radiation [32-34]. Thus, we initially hypothesized that knockdown of SFRP1 might result in increased Wnt signaling activity, thereby promoting tumor-associated properties of cells. This would be in line with previously published data showing reduced xenograft growth after SFRP1 overexpression in breast cancer cells presumably due to blockade of canonical Wnt signaling activity [35].

Surprisingly, however, we were neither able to detect Wnt activation by TOPFlash luciferase assays, changes in cellular localization of β-Catenin, nor detect any significant upregulation of known Wnt target genes. Therefore, we propose a different mechanism at which SFRP1 influences tumorigenic properties like invasion potential or resistance to chemotherapeutic agents. However, a Wnt dependent effect may also occur in triple negative cancers as in vivo Wnt signals may be supplied from the tumor stroma. Thus, Wnt dependent effects may also be contributing to the in vivo responsiveness of triple negative breast cancer patients to chemotherapy.

In addition to its known role in Wnt signaling, recent reports also demonstrate novel roles for SFRP1 signaling. One report showed binding of SFRP1 to thrombospondin-1, thereby inhibiting cancer cell adhesion and migration. This binding was conducted via the netrin related motif of SFRP1 [36]. These data are in accordance with our observation of increased invasiveness of SFRP1-depleted cells. Another study demonstrated an increased sensitivity of cells to TGF-β signaling upon SFRP1 reduction [37]. The TGF-β pathway is involved in epithelial to mesenchymal transition (EMT) as well as cellular migration in later stage mammary tumors, despite its known function as a tumor suppressor in early stage malignancies [38-40]. However, as expression of a majority of known EMT related genes are not substantially altered upon SFRP1 knockdown (Figure 4E), EMT may be of minor relevance in our experimental system. Recently, another study demonstrated a link between reduction of SFRP1 and reduction of apoptosis in vitro [41]. By using global gene expression profiles after SFRP1 knockdown, gene ontology analyses revealed upregulation of genes involved in migration processes, whereas genes involved in the positive regulation of apoptosis were downregulated (Tables 4, 5). Thus, chemotherapy might be reinforced by inhibition of apoptosis after SFRP1 knockdown. This view is supported by our observation of a slight decrease of apoptotic events upon SFRP1 depletion (Figure 3C, D). Apparently, pathways different from Wnt signaling presumably regulate processes that lead to increase of tumorigenic properties of cancer cells.

## Conclusions

Our study sheds light on the complex regulatory network of mammary tumorigenesis and tumor progression, proposing a model in which SFRP1 regulates either invasive processes via canonical Wnt signaling but also via different pathways, e.g. TGF-β signaling as well as apoptotic processes via so far unknown mechanisms (Figure 4F).

Furthermore, we conclude that SFRP1 might be clinically used to stratify patients, which suffer from triple negative breast cancer for responding to neoadjuvant chemotherapy. As the reduction of SFRP1 is in line with increased aggressiveness of cancer cells, its overexpression might be an approach to explore novel therapeutic projections [35]. Thus, an increased level of SFRP1 might sensitize triple negative breast cancer patients towards chemotherapy, thereby improving prognosis of this aggressive breast cancer subtype. Nevertheless, future analyses have to be undertaken to explore the role of SFRP1 in regulating mammary tumor progression, particularly progression of triple negative breast cancer.

## Methods

### Cell culture, chemicals

The human mammary epithelial cell line MCF10a and the cancerous cell lines HCC 1937, MDA-MB 468, BT-20, MDA-MB 453, HCC 1806, MDA-MB 231, SKBR-3 and MCF-7 were supplied from ATCC and cultured under recommended conditions. Medium, trypsin-EDTA, PBS, fetal calf serum and horse serum were received from PAA Laboratories.

In order to chemically stimulate Wnt signaling, cells were starved in medium without serum for 24 h followed by incubation with 10 mM LiCl (SIGMA Aldrich), which inhibits GSK3β, thereby activating Wnt signaling [19,21].

### Microarray gene expression analyses

A published microarray dataset was used for differential gene expression analysis [18]. For gene expression analysis in patients, triple negative breast cancer was defined using clinical measurements for ER, PR and HER2 as described previously and compared to the remaining cases merged as non-TNBC. Response to neoadjuvant chemotherapy was defined as absence of invasive breast cancer cells at the time of definitive surgery [42] and dichotomized as either pCR (n = 34) or residual invasive disease (RD; n = 99). Among cases with TNBC, 13 cases had pCR and 14 cases had RD. Gene expression data was processed and normalized using the robust multiarray average normalization algorithm as implemented in R-package affy version 1.32.0 [43].

Differential gene expression between patient subgroups was assessed using Welch's t statistic. The resulting p values were adjusted for control of the false discovery rate (FDR) according to Benjamini and Hochberg's method [44]. Analyses were performed in R using the Bioconductor multitest package version 2.10.0 [45].

For microarray experiments after SFRP1 knockdown in MDA-MB-468 cells, mRNA was converted into cDNA by using Superscript Double-Stranded cDNA Synthesis Kit (Invitrogen) according to manufactures protocol. Fluorescence labeling was performed using NimbleGen One-Color DNA Labeling Kit followed by hybridization onto arrays (NimbleGen human gene expression 12 × 135k arrays) according to protocol. By using DEVA software raw data was extracted. Further normalization was performed using GeneSpring Software. Normalized values were imported into gene ontology database DAVID (http://david.abcc.ncifcrf.gov; [26].

For expression analysis Wnt target genes were identified using the Wnt Homepage (http://www.stanford.edu/group/nusselab/cgi-bin/wnt/target_genes).

### Western Blot analysis

Cells were incubated with RIPA buffer (10 mM NaF, 1 mM Na_3_VO_4_, 10 mM β-Glycerophosphate, 7.6 mM Tris pH 7.4, 52 mM NaCl, 0.4% Triton X-100, 0.8 mM EDTA, proteinase inhibitor (SIGMA Aldrich). Protein quantification was performed via BCA assay (Pierce) according to the manufacturer’s protocol. SDS page electrophoresis and blotting were performed using standard protocols. Detection was performed using SFRP1 antibody (SIGMA Aldrich, SAB2900383) and β-Actin antibody (BioLegend, clone # 2 F1-1) and SuperSignal West Pico Chemiluminescent Substrate (Pierce). Bands were visualized with AGFA developer and fixer (AGFA).

### Quantitative real-time PCR

RNA isolation was performed using NucleoSpin RNA Kits (Macherey-Nagel) with on-column DNase digestion. Reverse transcription for real-time quantitative polymerase chain reaction (RT-qPCR) was performed using MMLV reverse transcriptase (USB (Affymetrix)) and Oligo-dT_15_ priming at 42°C for 1 hour and at 60°C for 10 minutes. A cDNA equivalent of 50 ng total RNA was used as template in a total reaction volume of 20 μl with Power SYBR Green PCR mix (Invitrogen) on an Step One Plus cycler (ABI). Primers were added at 0.375 μM each. Calculations were based on the ΔΔCt method using two housekeeping genes for normalization. Real time primer sequences can be found in supplemental Table 1 (Additional file 1: Table S1).

### siRNA mediated mRNA knockdown

siRNA mediated knockdown assays were implemented using SFRP1 siRNA (part no 4392422) and negative control siRNA (part no 4390844) (Applied Biosystems) in combination with DharmaFECT (ThermoScientific) transfection reagent according to the manufacturer’s protocol. Efficacy of knockdown was analyzed by qPCR and Western blotting 48 h – 72 h after transfection.

### Cell migration / invasion assay

For migration assays, cell culture inserts equipped with 8 μm membranes were used (Falcon). For invasion assays, BioCoat Matrigel invasion chambers (BD Biosciences) were used according to the manufacturer’s protocols. Briefly, 24 – 48 h after transfection, 2 – 5 ×10^4^ cells were seeded into cell culture inserts in medium without serum. The lower chamber was filled with medium containing serum as chemoattractant. 48 – 96 h after seeding cells, which passed the membranes, were fixed and stained using Diff-Quik staining set according to manufacturer’s protocol (Medion Diagnostics). Stained filters were mounted on microscope slides with VitroClud (Langenbrinck). Quantitative analysis was done by cell counting using Image J software.

### Luciferase assay

Luciferase assays were performed using TOPFlash or FOPFlash plasmids (addgene plasmid numbers 1256 and 12457, respectively) along with renilla normalization construct (pRL-TK, Promega) using Lipofectamine 2000 (Invitrogen). Luciferase constructs were transfected 48 h after siRNA transfection. Starvation medium as well as normal medium was changed the next day. After additional 24 h, cells were harvested and processed according to the DualGlo Luciferase protocol (Promega). Relative Luciferase activity was normalized to the activity of the FOPFlash mutant vector control.

### Immunohistochemistry/Immunocytochemistry

The study was approved by the local ethical review committee (Research ethics committee of the Medical Association Westfalen-Lippe and Westphalian Wilhelms University; ethical vote: 2013-156-f-S). We used tissue microarrays of 362 patients. Of these, 37 were diagnosed as being TNBC by missing expression of ER, PR and HER2. Immunohistochemistry of formalin-fixed, paraffin-embedded tissue microarrays was performed using primary antibody (SFRP1, Epitomics, clone# EPR7003) and biotinylated secondary antibodies (DAKO). Detection was performed using Chromogen Red (DAKO) and H&E (Merck). Slides were embedded with Scientific Cytoseal (Thermo Scientific Fisher).

For immunocytochemistry, cells were fixed with phosphate buffered formalin. Cells were blocked with 10% Aurion (DAKO) in PBS for 1 h. Cells were washed and incubated with primary antibody (β-Catenin, Cell Signaling, # 9587) diluted with Dako REALTM Antibody Diluent (overnight at 4°C). Fluorescent visualization was carried out using suitable Alexa Fluor-conjugated secondary antibody (1:600) together with 4′,6-diamidino-2-phenylindole (1:400) in in Dako REALTM Antibody Diluent) for 1 h at RT.

### Chemotherapy sensitivity assay

For analysis of chemotherapy sensitivity, cells were incubated with cytotoxic agents using decreasing concentrations: paclitaxel (10 pM - 1 μM), doxorubicin hydrochloride (500 pM - 50 μM), cis-diamineplatinum II dichloride (50 nM - 5 mM). After 96 hours, cell viability was determined via MTT (Thiazolyl Blue Tetrazolium Bromide) (all substances were received from SIGMA Aldrich) according to the manufacturer’s protocol. Measurements were performed at least in triplicates. Significance was calculated via one-side Welch’s t-test.

### Flow cytometry

Following transfection cells were stained for apoptosis as well as apoptosis/necrosis using the annexin V test kit from Becton Dickinson (San José, USA). Flow cytometric cell analysis and quantification of cell death took place on a flow cytometer (CyFlow Space, Partec, Germany) as described previously [46,47].

### Consent

Written informed consent was obtained from the patients for the publication of this report and any accompanying images.

## Abbreviations

SFRP1: Secreted frizzled receptor protein 1; HER2: Human epidermal growth factor receptor 2; TNBC: Triple negative breast cancer; siRNA: Small interfering RNA; ER: Estrogen receptor; PR: Progesterone receptor; PCR: Polymerase chain reaction; LiCl: Lithium chloride; pCR: Pathologic complete response; IHC: Immunohistochemistry; FISH: Fluorescence in situ hybridization; RD: Residual disease; EMT: Epithelial to mesenchymal transition.

## Competing interests

The authors declare no conflict of interests.

## Authors’ contribution

C.B. conception and design, collection and/or assembly of data, data analysis and interpretation, manuscript writing, final approval of manuscript, C.H. conception and design, collection and/or assembly of data, data analysis and interpretation, manuscript writing; C.R. data analysis and interpretation; S.S. and L.B. provision of study materials; G.H. and M.G. data analysis and interpretation; B.G. and P.J.B. collection and/or assembly of data, data analysis and interpretation; L.K. conception and design, financial support; C.L.: conception and design, data analysis and interpretation. All authors read and approved the final manuscript.

## Supplementary Material

Additional file 1: Table S1 Sequences of qPCR primers used in this study. Click here for file

## References

[B1] Carey L Winer E Viale G Cameron D Gianni L Triple-negative breast cancer: disease entity or title of convenience? Nat Rev Clin Oncol 2010 7 683 692 20877296 10.1038/nrclinonc.2010.154

[B2] Gluz O Liedtke C Gottschalk N Pusztai L Nitz U Harbeck N Triple-negative breast cancer–current status and future directions Ann Oncol 2009 20 1913 1927 19901010 10.1093/annonc/mdp492

[B3] Rakha EA El-Sayed ME Green AR Lee AHS Robertson JF Ellis IO Prognostic markers in triple-negative breast cancer Cancer 2007 109 25 32 17146782 10.1002/cncr.22381

[B4] Miller K Wang M Gralow J Dickler M Cobleigh M Perez EA Shenkier T Cella D Davidson NE Paclitaxel plus bevacizumab versus paclitaxel alone for metastatic breast cancer N Engl J Med 2007 357 2666 2676 18160686 10.1056/NEJMoa072113

[B5] Santana-Davila R Perez EA Treatment options for patients with triple-negative breast cancer J Hematol Oncol 2010 3 42 20979652 10.1186/1756-8722-3-42 PMC2987865

[B6] Cheang MCU Voduc D Bajdik C Leung S McKinney S Chia SK Perou CM Nielsen TO Basal-like breast cancer defined by five biomarkers has superior prognostic value than triple-negative phenotype Clin Cancer Res 2008 14 1368 1376 18316557 10.1158/1078-0432.CCR-07-1658

[B7] Dent R Trudeau M Pritchard KI Hanna WM Kahn HK Sawka CA Lickley LA Rawlinson E Sun P Narod SA Triple-Negative Breast Cancer: Clinical Features and Patterns of Recurrence Clin Cancer Res 2007 13 4429 4434 17671126 10.1158/1078-0432.CCR-06-3045

[B8] Ruijter TC Veeck J Hoon JPJ Engeland M Tjan-Heijnen VC Characteristics of triple-negative breast cancer J Cancer Res Clin Oncol 2010 137 183 192 21069385 10.1007/s00432-010-0957-x PMC3018596

[B9] Liedtke C Mazouni C Hess KR Andre F Tordai A Mejia JA Symmans WF Gonzalez-Angulo AM Hennessy B Green M Cristofanilli M Hortobagyi GN Pusztai L Response to Neoadjuvant Therapy and Long-Term Survival in Patients With Triple-Negative Breast Cancer J Clin Oncol 2008 26 1275 1281 18250347 10.1200/JCO.2007.14.4147

[B10] Kawano Y Kypta R Secreted antagonists of the Wnt signalling pathway J Cell Sci 2003 116 2627 2634 12775774 10.1242/jcs.00623

[B11] Caldwell GM Jones C Gensberg K Jan S Hardy RG Byrd P Chughtai S Wallis Y Matthews GM Morton DG The Wnt antagonist sFRP1 in colorectal tumorigenesis Cancer Res 2004 64 883 888 14871816 10.1158/0008-5472.can-03-1346

[B12] Fukui T Kondo M Ito G Maeda O Sato N Yoshioka H Yokoi K Ueda Y Shimokata K Sekido Y Transcriptional silencing of secreted frizzled related protein 1 (SFRP 1) by promoter hypermethylation in non-small-cell lung cancer Oncogene 2005 24 6323 6327 16007200 10.1038/sj.onc.1208777

[B13] Lodygin D Epanchintsev A Menssen A Diebold J Hermeking H Functional epigenomics identifies genes frequently silenced in prostate cancer Cancer Res 2005 65 4218 4227 15899813 10.1158/0008-5472.CAN-04-4407

[B14] Suzuki H Watkins DN Jair K-W Schuebel KE Markowitz SD Dong Chen W Pretlow TP Yang B Akiyama Y van Engeland M Toyota M Tokino T Hinoda Y Imai K Herman JG Baylin SB Epigenetic inactivation of SFRP genes allows constitutive WNT signaling in colorectal cancer Nat Genet 2004 36 417 422 15034581 10.1038/ng1330

[B15] Takada T Yagi Y Maekita T Imura M Nakagawa S Tsao S-W Miyamoto K Yoshino O Yasugi T Taketani Y Ushijima T Methylation-associated silencing of the Wnt antagonist SFRP1 gene in human ovarian cancers Cancer Sci 2004 95 741 744 15471560 10.1111/j.1349-7006.2004.tb03255.x PMC11160044

[B16] Veeck J Niederacher D An H Klopocki E Wiesmann F Betz B Galm O Camara O Dürst M Kristiansen G Huszka C Knüchel R Dahl E Aberrant methylation of the Wnt antagonist SFRP1 in breast cancer is associated with unfavourable prognosis Oncogene 2006 25 3479 3488 16449975 10.1038/sj.onc.1209386

[B17] Yang Z-Q Liu G Bollig-Fischer A Haddad R Tarca AL Ethier SP Methylation-associated silencing of SFRP1 with an 8p11-12 amplification inhibits canonical and non-canonical WNT pathways in breast cancers Int J Cancer 2009 125 1613 1621 19569235 10.1002/ijc.24518 PMC2735097

[B18] Hess KR Anderson K Symmans WF Valero V Ibrahim N Mejia JA Booser D Theriault RL Buzdar AU Dempsey PJ Rouzier R Sneige N Ross JS Vidaurre T Gómez HL Hortobagyi GN Pusztai L Pharmacogenomic Predictor of Sensitivity to Preoperative Chemotherapy With Paclitaxel and Fluorouracil, Doxorubicin, and Cyclophosphamide in Breast Cancer J Clin Oncol 2006 24 4236 4244 16896004 10.1200/JCO.2006.05.6861

[B19] Klein PS Melton DA A molecular mechanism for the effect of lithium on development Proc Natl Acad Sci U S A 1996 93 8455 8459 8710892 10.1073/pnas.93.16.8455 PMC38692

[B20] Ng SS Mahmoudi T Danenberg E Bejaoui I de Lau W Korswagen HC Schutte M Clevers H Phosphatidylinositol 3-Kinase Signaling Does Not Activate the Wnt Cascade J Biol Chem 2009 284 35308 35313 19850932 10.1074/jbc.M109.078261 PMC2790960

[B21] O'Brien WT Harper AD Jové F Woodgett JR Maretto S Piccolo S Klein PS Glycogen synthase kinase-3beta haploinsufficiency mimics the behavioral and molecular effects of lithium J Neurosci 2004 24 6791 6798 15282284 10.1523/JNEUROSCI.4753-03.2004 PMC5328671

[B22] Arnsdorf EJ Tummala P Jacobs CR Non-Canonical Wnt Signaling and N-Cadherin Related β-Catenin Signaling Play a Role in Mechanically Induced Osteogenic Cell Fate PLoS One 2009 4 e5388 19401766 10.1371/journal.pone.0005388 PMC2670536

[B23] Liu Y Rubin B Bodine PVN Billiard J Wnt5a induces homodimerization and activation of Ror2 receptor tyrosine kinase J Cell Biochem 2008 105 497 502 18615587 10.1002/jcb.21848

[B24] Luga V Zhang L Viloria-Petit AM Ogunjimi AA Inanlou MR Chiu E Buchanan M Hosein AN Basik M Wrana JL Exosomes Mediate Stromal Mobilization of Autocrine Wnt-PCP Signalingin Breast Cancer Cell Migration Cell 2012 151 1542 1556 23260141 10.1016/j.cell.2012.11.024

[B25] Lamouille S Xu J Derynck R Molecular mechanisms of epithelial– mesenchymal transition Nat Rev Mol Cell Biol 2014 15 178 196 24556840 10.1038/nrm3758 PMC4240281

[B26] Huang DW Sherman BT Lempicki RA Systematic and integrative analysis of large gene lists using DAVID bioinformatics resources Nat Protoc 2009 4 44 57 19131956 10.1038/nprot.2008.211

[B27] Neve RM Chin K Fridlyand J Yeh J Baehner FL Fevr T Clark L Bayani N Coppe J-P Tong F Speed T Spellman PT DeVries S Lapuk A Wang NJ Kuo W-L Stilwell JL Pinkel D Albertson DG Waldman FM McCormick F Dickson RB Johnson MD Lippman M Ethier S Gazdar A Gray JW A collection of breast cancer cell lines for the study of functionally distinct cancer subtypes Cancer Cell 2006 10 515 527 17157791 10.1016/j.ccr.2006.10.008 PMC2730521

[B28] Bauer JA Chakravarthy AB Rosenbluth JM Mi D Seeley EH De Matos Granja-Ingram N Olivares MG Kelley MC Mayer IA Meszoely IM Means-Powell JA Johnson KN Tsai CJ Ayers GD Sanders ME Schneider RJ Formenti SC Caprioli RM Pietenpol JA Identification of markers of taxane sensitivity using proteomic and genomic analyses of breast tumors from patients receiving neoadjuvant paclitaxel and radiation Clin Cancer Res 2010 16 681 690 20068102 10.1158/1078-0432.CCR-09-1091 PMC2892225

[B29] Lehmann BD Bauer JA Chen X Sanders ME Chakravarthy AB Shyr Y Pietenpol JA Identification of human triple-negative breast cancer subtypes and preclinical models for selection of targeted therapies J Clin Invest 2011 121 2750 2767 21633166 10.1172/JCI45014 PMC3127435

[B30] Jiang W Freidlin B Simon R Biomarker-adaptive threshold design: a procedure for evaluating treatment with possible biomarker-defined subset effect JNCI J of the National Cancer Institute 2007 99 1036 1043 10.1093/jnci/djm022 17596577

[B31] Bovolenta P Esteve P Ruiz JM Cisneros E Lopez-Rios J Beyond Wnt inhibition: new functions of secreted Frizzled-related proteins in development and disease J Cell Sci 2008 121 737 746 18322270 10.1242/jcs.026096

[B32] Clevers H Nusse R Wnt/b-Catenin Signaling and Disease Cell 2012 149 1192 1205 22682243 10.1016/j.cell.2012.05.012

[B33] Holland JD Klaus A Garratt AN Birchmeier W Wnt signaling in stem and cancer stem cells Curr Opin Cell Biol 2013 25 2 254 264 23347562 10.1016/j.ceb.2013.01.004

[B34] Klaus A Birchmeier W Wnt signalling and its impact on development and cancer Nat Rev Cancer 2008 8 387 398 18432252 10.1038/nrc2389

[B35] Matsuda Y Schlange T Oakeley EJ Boulay A Hynes NE WNT signaling enhances breast cancer cell motility and blockade of the WNT pathway by sFRP1 suppresses MDA-MB-231 xenograft growth Breast Cancer Res 2009 11 R32 19473496 10.1186/bcr2317 PMC2716500

[B36] Martin-Manso G Calzada MJ Chuman Y Sipes JM Xavier CP Wolf V Kuznetsova SA Rubin JS Roberts DD sFRP-1 binds via its netrin-related motif to the N-module of thrombospondin-1 and blocks thrombospondin-1 stimulation of MDA-MB-231 breast carcinoma cell adhesion and migration Arch Biochem Biophys 2011 509 147 156 21402050 10.1016/j.abb.2011.03.004 PMC3085965

[B37] Gauger KJ Chenausky KL Murray ME Schneider SS SFRP1 reduction results in an increased sensitivity to TGF-β signaling BMC Cancer 2011 11 59 21303533 10.1186/1471-2407-11-59 PMC3041779

[B38] Muraoka RS Dumont N Ritter CA Dugger TC Brantley DM Chen J Easterly E Roebuck LR Ryan S Gotwals PJ Koteliansky V Arteaga CL Blockade of TGF-beta inhibits mammary tumor cell viability, migration, and metastases J Clin Invest 2002 109 1551 1559 12070302 10.1172/JCI15234 PMC151012

[B39] Muraoka-Cook RS Shin I Yi JY Easterly E Barcellos-Hoff MH Yingling JM Zent R Arteaga CL Activated type I TGFbeta receptor kinase enhances the survival of mammary epithelial cells and accelerates tumor progression Oncogene 2006 25 3408 3423 16186809 10.1038/sj.onc.1208964

[B40] Roberts AB Wakefield LM The two faces of transforming growth factor beta in carcinogenesis Proc Natl Acad Sci U S A 2003 100 8621 8623 12861075 10.1073/pnas.1633291100 PMC166359

[B41] Gauger KJ Schneider SS Tumour supressor secreted frizzled related protein 1 regulates p53-mediated apoptosis Cell Biol Int 2013 38 124 130 24038862 10.1002/cbin.10176

[B42] Mazouni C Peintinger F Wan-Kau S Andre F Gonzalez-Angulo AM Symmans WF Meric-Bernstam F Valero V Hortobagyi GN Pusztai L Residual ductal carcinoma in situ in patients with complete eradication of invasive breast cancer after neoadjuvant chemotherapy does not adversely affect patient outcome J Clin Oncol 2007 25 2650 2655 17602071 10.1200/JCO.2006.08.2271

[B43] Irizarry RA Hobbs B Collin F Beazer-Barclay YD Antonellis KJ Scherf U Speed TP Exploration, normalization, and summaries of high density oligonucleotide array probe level data Biostatistics 2003 4 249 264 12925520 10.1093/biostatistics/4.2.249

[B44] Benjamini Y Drai D Elmer G Kafkafi N Golani I Controlling the false discovery rate in behavior genetics research Behav Brain Res 2001 125 279 284 11682119 10.1016/s0166-4328(01)00297-2

[B45] Gentleman RC Carey VJ Bates DM Bolstad B Dettling M Dudoit S Ellis B Gautier L Ge Y Gentry J Hornik K Hothorn T Huber W Iacus S Irizarry R Leisch F Li C Maechlers M Rossini AJ Sawitzki G Smith C Smyth G Tierney L Yang JYH Zhang J Bioconductor: open software development for computational biology and bioinformatics Genome Biol 2004 5 R80 15461798 10.1186/gb-2004-5-10-r80 PMC545600

[B46] Greve B Dreffke K Rickinger A Könemann S Fritz E Eckardt-Schupp F Amler S Sauerland C Braselmann H Sauter W Illig T Schmezer P Gomolka M Willich N Bölling T Multicentric investigation of ionising radiation-induced cell death as a predictive parameter of individual radiosensitivity Apoptosis 2009 14 226 235 19142732 10.1007/s10495-008-0294-6

[B47] Greve B Kelsch R Spaniol K Eich HT Götte M Flow cytometry in cancer stem cell analysis and separation Cytometry A 2012 81 284 293 22311742 10.1002/cyto.a.22022

