# Supplementary material for: Influence of secreted frizzled receptor protein 1 (SFRP1) on neoadjuvant chemotherapy in triple negative breast cancer does not rely on WNT signaling
Source: Mol Cancer. 2014 Jul 17;13:174. doi: 10.1186/1476-4598-13-174 (PMC4110378; doi:10.1186/1476-4598-13-174)
Supplement: Additional file 1: Table S1 — Sequences of qPCR primers used in this study. [file 1476-4598-13-174-S1.pdf]

Table S1: Sequences of qPCR primers used in this study

| | ffw | rev |
|---|---|---|
| ACTB | TCAAGATCATTGCTCCTCCTGAG | ACATCTGCTGGAAGGTGGACA |
| AXIN2 | TTGGACCACAGCCATTCAGG | GGAAAGGTGCTGCTGGGTCT |
| CCND1 | GTCCTGTGACGCGCAAGTCT | ACATGTTGGTGCTGGGAAGC |
| CDH1 | CAAAGCCCAGAATCCCCAAG | CACACCTGGAATTGGGCAAA |
| CDH2 | TTCTGACAACAGCTTTGCCTCTG | TTTATTCAGAACGCTGGGGTCA |
| DAAM1 | GACAGGTGGAGGGAAAGTCACA | CCACCAGGATGGCCATATCA |
| DAAM2 | TCACCTGAAGGTGCCAAGGA | TGGGAGGACCAGAAGGAACC |
| DSP | AGGCTGCAAGACACCAGCAG | AAGGCGCAGCCCAGTGATA |
| ELF5 | TGACTGACAGCTAGGTGGACTGC | GCGATTCAGTGCCTCACCAC |
| FN | CCAAGCATCACCCTGGGAGT | CGAAGCAGAACAGGCAATGTG |
| FOXC2 | GCGGCCTCCTGGTATCTCAA | AGCCGGTGGGAGTTGAACAT |
| FZD7 | GCTGACCCTGTCTCTGTGTGGT | GCCCATGGTTCAAACCTTCC |
| MAPK | GCAAACAAGAATGCCAGTGCTC | AGGTGGGTGCAGTTCCCTGT |
| MMP2 | GCCGTGTTTGCCATCTGTTT | CTGCAGGGAGCAGAGATTCG |
| MMP9 | ATTTCTGCCAGGACCGCTTC | CTCAGGGCACTGCAGGATGT |
| MUC1 | GCAGGTAATGGTGGCAGCAG | GTGGAGTGGAATGGCACTGG |
| MYC | CGGAACTCTTGTGCGTAAGGAA | GCCAAGGTTGTGAGGTTGCAT |
| NKD1 | TCCACATCCCACACCGAAAG | TCTGAGACCTTGGCGATTGG |
| OCLN | CCACTGGCTGAGCTGATTGTG | TCTGTTCTTTGCCCATGAGTGG |
| PRICKLE1 | GAAAGTCCCGCTCCGACAAT | ACAGCCGCAGTCTGTCCTTG |
| PRICKLE2 | GCAGTTTCAGATCGCCACCTT | TGAGGCACCAGAGGGACAAA |
| PRICKLE3 | GCAGCCTCCACAGCCTCTTT | TCTGTGCTGGTGCCTTTGGT |
| PRICKLE4 | GAACCGCTACTCGGATGCAG | CACCGAGGTTTGGTCCCTTC |
| RAC1 | CGCCTCCTGTAGTCGCTTTG | CACGCTGTATTCTCGCCAGTG |
| RHOA1 | GGTTTCACGCCTGAGGCAAT | GCCAGATGCTTAAGTCCAGGTG |
| ROCK2 | TGGCAACTGGGAAGAAGTGG | CGGCAAGAATGTCCTGCGTA |
| ROR2 | CACGTGCACATGAGGTCCAT | CACCGGGTGTGGGATTTACA |
| RPL37 | GTGGTTCCTGCATGAAGACAGTG | TTCTGATGGCGGACTTTACCG |
| RUNX2 | CCACACCATTAGGGACCATCTG | ACCTGGATTCTGGGCCAGTC |
| SFRP1 | GATGCAGGAGGCTCAGGTGAT | GCTGGCAACAGGTCAGAACG |
| SNAI1 | CGAGCCCAGGCAGCTATTTC | CCCGACAAGTGACAGCCATT |
| SNAI2 | ATCTGCCAGACGCGAACTCA | GGCAACCAGACAACCGACAT |
| SOX10 | GCATCGGAGGCCTTACCACT | TCTGGGTGGCCTAGCCCTTA |
| TCF7L2 | AAACCAGCTGCCGCTTTTATG | GCAACATCAACATGCCTAGGTTTT |

| | | |
|---|---|---|
| TJP | TGATACAATGCTGTGCCCTAAAGTG | GAAATCGTGCTGATGTGCCATAAT |
| TWIST | GCGGCCAGGTACATCGACTT | TGCAGCTTGCCATCTTGGAG |
| VANGL | CAACCAGCCTCTGCTCATGG | AGCCCACTACCCAGTTTATGCAG |
| VIM | TCAGCATCACGATGACCTTGAA | CTGCAGAAAGGCACTTGAAAGC |
| WNT3A | AGGTCCCACAGCCCTGAGAT | TCCAGGAAAGCGGACCATTT |
| WNT5A | TCGTTAGCAGCATCAGTCCACA | GACCTGTGCCTTCGTGCCTA |
| ZEB2 | TGGGCTAGTAGGCTGTGTCCA | TCATCTTCAACCCTGAAACAGAGG |
